# Evaluation of myocardial involvement in patients with connective tissue disorders: a multi-parametric cardiovascular magnetic resonance study

**DOI:** 10.1186/s12968-016-0288-4

**Published:** 2016-10-13

**Authors:** Agnes Mayr, Daniel Kitterer, Joerg Latus, Hannah Steubing, Joerg Henes, Francesco Vecchio, Philipp Kaesemann, Alexandru Patrascu, Andreas Greiser, Stefan Groeninger, Niko Braun, M. Dominik Alscher, Udo Sechtem, Heiko Mahrholdt, Simon Greulich

**Affiliations:** 1 Division of Radiology, University Hospital Innsbruck, Innsbruck, Austria; 2 Division of Nephrology, Department of Internal Medicine, Robert-Bosch-Medical Center Stuttgart, Stuttgart, Germany; 3 Division of Cardiology, Robert-Bosch-Medical Center Stuttgart, Auerbachstrasse 110, 70376 Stuttgart, Germany; 4 Centre for Interdisciplinary Clinical Immunology, Rheumatology and Auto-inflammatory Diseases and Department of Internal Medicine II (Oncology, Haematology, Immunology, Rheumatology, Pulmology), University Hospital Tuebingen, Tuebingen, Germany; 5 Siemens Healthcare GmbH, Erlangen, Germany

**Keywords:** Connective tissue disorders, Myocardial involvement, CMR, LGE, Mapping

## Abstract

**Background:**

Severe arrhythmias or heart failure may be surrogates of myocardial involvement in patients with connective tissue disorders (CTD). However, most patients present with unspecific symptoms, normal ECG, and preserved left ventricular ejection fraction (LV-EF). Therefore, timely diagnosis by an accurate technique is crucial. Late gadolinium enhancement (LGE) cardiovascular magnetic resonance (CMR) has proven value for the detection of focal processes, but due to the often diffuse character of fibrosis/inflammation in CTD patients, CMR mapping techniques might be of incremental value for the assessment of myocardial involvement. Purpose of this study was to evaluate a multi-parametric CMR protocol as a screening tool for myocardial involvement in CTD patients.

**Methods:**

Forty CTD patients were prospectively enrolled and underwent CMR, twenty healthy volunteers served as control group.

**Results:**

Mean LV-EF was 62 %; LGE prevalence was low (18 %). CTD patients had higher native T1 (1008 vs. 962 ms, *p* = 0.001), lower post contrast T1 (494 vs. 526 ms, *p* = 0.008), expanded extracellular volume (ECV) (28 vs. 25 %, *p* = 0.001), and higher T2 values (53 vs. 49 ms, *p* < 0.001) compared to controls. Among patients with values higher than the 95 % percentile of healthy controls, native T1 and T2 values seem to be the most promising discriminators.

**Conclusion:**

CTD patients showed higher T1, ECV, and T2 values compared to controls, with most significant differences for native T1 and T2, which seem to be independent of the presence of LGE. Our data suggest that CMR mapping techniques are of incremental value in the detection of myocardial involvement in CTD patients.

**Electronic supplementary material:**

The online version of this article (doi:10.1186/s12968-016-0288-4) contains supplementary material, which is available to authorized users.

## Background

Connective tissue disorders (CTD) are a heterogeneous form of rheumatic disorders comprising systemic lupus erythematosus (SLE), systemic sclerosis (SSc), Sjögren’s syndrome, inflammatory muscle diseases and overlap syndrome [1]. There is a high variety in the prevalence of CTD, which may occur at all ages, but show a higher prevalence in young adults [1]. SLE is one of the most common autoimmune disorders in the western world with a prevalence ranging from 15 to 50 per 100,000 persons [1]. Cardiovascular complications may manifest as inflammation of valves, myocardium, pericardium resulting in myocardial dysfunction, and heart failure [2]. The prevalence of SSc is estimated about 26 per 100,000 persons [1]. SSc is characterized by structural and functional abnormalities of small blood vessels, fibrosis of the skin and internal organs, activation of the immune system and autoimmunity [1]. Myocardial involvement often remains subclinical, however autopsy studies reveal diffuse myocardial fibrosis in up to 80 % of cases [3–6], and sudden cardiac death occurs in up to 21 % of SSc patients [5]. Therefore, timely detection of myocardial involvement in stages, which might be potentially reversible by an adequate treatment regimen, is of high clinical interest in patients with CTD.

Cardiovascular magnetic resonance (CMR) offers beside functional assessment excellent tissue characterization without the need of radiation. Recent data suggest that a CMR approach, including late gadolinium enhancement (LGE) for the detection of focal fibrosis, and T1 mapping sequences for the detection of diffuse fibrosis, might be useful in the detection of myocardial involvement in patients with SLE and SSc [2, 3]. However, for the assessment of inflammation, these groups used standard T2-weighted images, which are known for severe limitations (e.g. proneness for artifacts) [7]. In the meantime, new T2 mapping sequences were developed, overcoming most of the standard T2-weighting limitations [8].

Consequently, aim of our study was to evaluate a comprehensive CMR protocol, including LGE and quantitative T1 and T2 mapping techniques for the assessment of both fibrosis and inflammation, as a screening tool for potential myocardial involvement in patients presenting with CTD.

## Methods

### Patient population

Forty patients presenting at our institution between October 2013 and March 2016 were consecutively enrolled if they fulfilled the following criteria: 1) connective tissue disorder; and 2) no history of CAD, myocardial infarction and/or prior revascularization; and 3) successfully underwent CMR imaging. Exclusion criteria were contraindications for CMR (e.g. pregnancy, pacemaker/ICD, glomerular filtration rate <30 ml/min., previous adverse reactions to gadolinium, cochlea implant).

Healthy volunteers (*n* = 20) with no history of cardiac disease and free of symptoms served as control group. Prior to CMR, all participants provided a blood sample for measurement of hematocrit. The ethics committee of the University of Tuebingen approved the study and all patients gave written informed consent.

### CMR protocol

ECG-gated CMR was performed in breath-hold using a 1.5 T Magnetom Aera (Siemens Healthcare, Erlangen, Germany) in line with current recommendations [9]. Both cine and LGE short axis images were prescribed every 10 mm (slice thickness 6 mm) from base to apex. In-plane resolution was typically 1.2 × 1.8 mm. Cine was performed using a steady-state free-precession (SSFP) sequence. LGE images were acquired on average 5–10 min after contrast using a segmented inversion recovery gradient echo (IR-GRE)-sequence constantly adjusting inversion time to null normal myocardium [10, 11]. The contrast dose (Gadopentetate-Dimeglumine) was 0.15 mmol/kg.

A modified look-locker inversion recovery prototype sequence (MOLLI) was used for T1 mapping and performed in a single midventricular short-axis (SAX) slice at mid-diastole, prior to and 20 min after administration of contrast, in line with current recommendations [12, 13].

Short axis T2 mapping was performed in a matching midventricular SAX before administration of contrast agent using an ECG-triggered T2-prepared single-shot bSSFP prototype sequence with multiple T2 preparation times [8].

More detailed information on T1 and T2 mapping sequences is provided in the Additional file 1.

### CMR analysis

Cine and LGE images were evaluated by experienced observers (S.G., H.M.) as described elsewhere [14]. In brief, endocardial and epicardial borders were outlined on the short-axis cine images. Volumes, mass and ejection-fraction were derived by summation of epicardial and endocardial contours. Extent of LGE was assessed using QMass software (Medis, Leiden, The Netherlands), and the results were expressed as percentage of myocardial mass. The distribution of LGE was characterized as epicardial, intramural, transmural, or subendocardial [14].

Color-coded T1, ECV, and T2 maps were generated based on inline-generated, motion corrected raw images using QMap software 1.0 (Medis, Leiden, the Netherlands) in a single matching midventricular SAX. Motion-corrected T1 maps were examined for quality in three modalities: 1) raw T1 images 2) T1 maps 3) R^2^ maps. Endo- and epicardial contours were manually drawn by two experienced observers (S.G., A.M.), and then divided into 6 segments using the anterior right ventricular insertion point as reference. Care was taken to avoid partial volume effects at the endocardial and epicardial borders for T1, ECV and T2 maps. Global T1, ECV, and T2 values were calculated: T1 values were determined by fitting an exponential model to the measured data [15]. Prior to CMR, the hematocrit was determined in all subjects, allowing with native and post contrast T1 measurements of the myocardium and blood pool the calculation of extracellular volume (ECV), using a previously described equation [16]. T2 results were obtained by fitting a 2-parameter intensity-weighted exponential model (no offset term) [17].

### Variables and definitions

All variables were collected directly from patients, and/or medical records except CMR parameters, which were evaluated as described above. Most variables are self-explanatory; all others are defined below.

Underlying connective tissue disorders had to fulfill the diagnostic criteria of the American College of Rheumatology or the European League Against Rheumatism, respectively. Due to the variety of CTD (*n* = 5), these were clustered into three subgroups:

1. SSc
2. SLE
3. “Others”: overlap syndrome, Sjögren’s syndrome, and polymyositis.

Evaluation of disease activity in SSc and SLE patients:

SSc: ESSG = European scleroderma study group [18].

SLE: SLEDAI = Systemic Lupus Erythematosus Disease Activity Index [19].

For SSc and SLE, subgroup analyses were performed. Due to the low number of patients and the heterogeneity of CTD in the “others” group (3 different CTD in 10 patients) no further subgroup analysis was performed.

### Statistical analysis

Absolute numbers and percentages were computed to describe the patient population. All continuous variables were tested for normality using the Kolmogorov-Smirnov test. Normally distributed continuous variables were expressed as means (with standard deviation) and skewed variables were presented as medians (with quartiles). Comparisons between groups were made using the Mann-Whitney *U* test or the Fisher’s exact test, as appropriate. P-values (two-tailed) of <0.05 were considered significant. All statistical analyses were performed using SPSS, version 22.0 (IBM Corp., Armonk, NY, USA).

## Results

### Patient characteristics

In total *n* = 60 subjects were included in the final analysis, see Table 1: *n* = 40 patients with CTD, *n* = 20 healthy individuals served as control group. At inclusion, CTD patients were 54 ± 17 years of age, predominantly female (87 %), and did not differ significantly from the control group, *p* = 0.10 for age and *p* = 0.27 for gender, respectively.

**Table 1:** Baseline characteristics

	All patients	SLE^a^	SSc^a^	Other CTD
	*n* = 40	*n* = 13	*n* = 17	*n* = 10
Age (yrs)	54 ± 17	45 ± 16	55 ± 16	62 ± 16
Gender (male)	5 (13 %)	3 (23 %)	1 (6 %)	1 (10 %)
Diagnosis				
Systemic lupus erythematosus	13 (33 %)	13 (100 %)	-	-
Systemic sclerosis	17 (38 %)	-	17 (100 %)	-
Overlap syndrome	6 (15 %)	-	-	6 (60 %)
Sjögren’s syndrome	3 (7 %)	-	-	3 (30 %)
Polymyositis	1 (2 %)	-	-	1 (10 %)
Cardiovascular risk factors				
Diabetes	1 (2 %)	-	-	1 (10 %)
Hypertension	13 (33 %)	4 (33 %)	4 (23 %)	5 (50 %)
Smoking^b^	14 (35 %)	6 (46 %)	7 (41 %)	1 (10 %)
Hyperlipidemia	7 (18 %)	1 (8 %)	2 (12 %)	4 (40 %)
Family history of CVD	14 (35 %)	6 (46 %)	4 (23 %)	4 (40 %)
Obesity (BMI ≥ 30 kg/m^2^)	4 (10 %)	2 (15 %)	2 (12 %)	-
Symptoms (multiple possible)				
Angina	9 (23 %)	3 (23 %)	2 (12 %)	4 (40 %)
Dyspnea	13 (33 %)	3 (23 %)	6 (35 %)	4 (40 %)
Palpitations	3 (7 %)	1 (8 %)	-	2 (20 %)
Syncope	1 (2 %)	-	-	1 (10 %)
ECG abnormality	8 (20 %)	1 (8 %)	3 (18 %)	4 (40 %)
Years since diagnosis				
< 1	8 (20 %)	5 (38.5 %)	1 (6 %)	2 (20 %)
1–4	12 (30 %)	2 (15 %)	9 (53 %)	1 (10 %)
5–9	6 (15 %)	1 (8 %)	3 (18 %)	2 (20 %)
≥ 10	14 (35 %)	5 (38.5 %)	4 (23 %)	5 (50 %)
Disease activity				
SLEDAI	-	16 (6–23)	-	-
ESSG	-	-	3.5 (1.4–5.5)	-
Hematocrit	0.38 (0.34–0.40)	0.38 (0.34–0.40)	0.38 (0.34–0.42)	0.38 (0.37–0.39)
Medication				
Beta-blockers	6 (15 %)	4 (31 %)	-	2 (20 %)
ARB	19 (48 %)	5 (39 %)	9 (53 %)	5 (50 %)
ASA	6 (15 %)	1 (8 %)	2 (12 %)	3 (30 %)
CCB	6 (15 %)	1 (8 %)	4 (23 %)	1 (10 %)
Statins	7 (18 %)	3 (23 %)	2 (12 %)	2 (20 %)
Diuretics	6 (15 %)	3 (23 %)	2 (12 %)	1 (10 %)
Steroids	24 (60 %)	10 (77 %)	8 (47 %)	6 (60 %)
NSAID	1 (2 %)	1 (8 %)	-	-
Chloroquines	4 (10 %)	2 (15 %)	1 (6 %)	1 (10 %)
Antibodies	1 (2 %)	1 (8 %)	-	-
Cyclophosphamide	9 (23 %)	5 (39 %)	3 (18 %)	1 (10 %)
Azathioprine	3 (8 %)	2 (15 %)	-	1 (10 %)
Methotrexate	1 (2 %)	-	-	1 (10 %)

All values are n (%) or mean ± SD or interquartile ranges

*SLE* systemic lupus erythematosus, *SSc* systemic sclerosis, *CTD* connective tissue disease, *CVD* cardiovascular disease, *BMI* body mass index, *ECG* electrocardiogram, *SLEDAI* systemic lupus erythematosus disease activity index, *ACR* American College of Rheumatology, *ESSG* European scleroderma study group, *ARB* angiotensin receptor blockers, *ASA* acetylsalicylic acid, *CCB* calcium channel blockers, *NSAID* nonsteroidal anti-inflammatory drug

^a^percentages based on number of SLE patients/SSc patients, respectively

^b^Current or ever-smokers

Most patients suffered from SSc (*n* = 17) or SLE (*n* = 13). Others (*n* = 10) had overlap syndrome (*n* = 6), Sjögren’s syndrome (*n* = 3), and polymyositis (*n* = 1). Nonspecific dyspnea and angina were the most frequently reported symptoms in the overall patient population (33 and 23 %, respectively). ECG abnormalities were detected in *n* = 8 (20 %) of the patients. In detail, *n* = 3 showed left bundle branch block (in all of them CAD could be excluded by coronary angiography), *n* = 2 had atrial fibrillation (one patient had coronary angiography and showed no CAD), *n* = 2 had ventricular extrasystoles (in one of the patients CAD was ruled out by coronary angiography), *n* = 1 patient showed a right bundle branch block. The majority (60 %) of our overall CTD population was on steroids during the time of CMR. Details are displayed in Table 1.

### General CMR results

CMR findings can be viewed in Table 2. The mean LV-EF was 62 %, and did not differ to our control group (*p* = 0.41). Furthermore, functional CMR parameters (LV size, mass, etc.) were not significantly different between CTD patients and controls. LGE was present in 7 (18 %) of the CTD patients, most commonly occurring in a non-ischemic pattern (epicardial and/or intramural) [14]. LGE was not present in any of the controls.

**Table 2:** CMR findings

	Controls (*n* = 20)	Patients (*n* = 40)	*P*
LV-EF (%)	66 ± 4	62 ± 12	0.41
LV-EDV (ml)	109 ± 28	108 ± 36	0.76
LV-ESV (ml)	37 ± 13	41 ± 25	0.86
LV-SV	71 ± 16	66 ± 13	0.41
LV-EDD	45 ± 5	46 ± 5	0.67
LA (cm^2^)	20 ± 4	20 ± 5	0.68
IVS (mm)	10 ± 2	9 ± 3	0.63
PA (mm)	24 ± 4	24 ± 6	0.56
LV mass (g)	76 ± 18	82 ± 24	0.50
LGE per patient	-	7 (18 %)	
Epicardial	-	1 (2 %)	
Intramural	-	6 (15 %)	
Transmural	-	1 (2 %)	
Subendocardial	-	-	
% LV mass	-	5.3	
Native T1 (ms)	962 (947–987)	1008 (990–1042)	**0.001**
Post contrast T1 (ms)	526 (508–553)	494 (477–522)	**0.008**
ECV (%)	25 (24–27)	28 (26–31)	**0.001**
T2 (ms)	49 (48–51)	53 (52–58)	**<0.001**

All values are n or mean ± SD or interquartile ranges

*CMR* cardiac magnetic resonance, *LV* left ventricular, *EF* ejection fraction, *EDV* end-diastolic volume, *ESV* end-systolic volume, *SV* stroke volume, *EDD* end-diastolic diameter, *LA* left atrium, *IVS* interventricular septum, *PA* pulmonary artery, *LGE* late gadolinium enhancement, *ECV* extracellular volume

Looking at the SSc and SLE subgroups (Tables 3 and 4) revealed that mean LV-EF was also preserved, and the prevalence of LGE tended to be low (12 % SSc, 23 % in SLE).

**Table 3:** CMR findings systemic sclerosis (SSc)

	Controls (*n* = 20)	Patients (*n* = 17)	*P*
LV-EF (%)	66 ± 4	62 ± 16	0.48
LV-EDV (ml)	109 ± 28	105 ± 29	0.82
LV-ESV (ml)	37 ± 13	38 ± 14	0.87
LV-SV	71 ± 16	67 ± 16	0.63
LV-EDD	45 ± 5	46 ± 4	0.42
LA (cm^2^)	20 ± 4	20 ± 5	0.93
IVS (mm)	10 ± 2	10 ± 4	0.76
PA (mm)	24 ± 4	26 ± 8	0.81
LV mass (g)	76 ± 18	81 ± 31	0.82
LGE per patient	-	2 (12 %)	
Epicardial	-	-	
Intramural	-	2 (12 %)	
Transmural	-	-	
Subendocardial	-	-	
% LV mass	-	1.3	
Native T1 (ms)	962 (947–987)	1031 (1007–1075)	**<0.001**
Post contrast T1 (ms)	526 (508–553)	494 (474–525)	**0.02**
ECV (%)	25 (24–27)	31 (28–34)	**<0.001**
T2 (ms)	49 (48–51)	58 (54–59)	**<0.001**

All values are n or mean ± SD or interquartile ranges

*CMR* cardiac magnetic resonance, *LV* left ventricular, *EF* ejection fraction, *EDV* end-diastolic volume, *ESV* end-systolic volume, *SV* stroke volume, *EDD* end-diastolic diameter, *LA* left atrium, *IVS* interventricular septum, *PA* pulmonary artery, *LGE* late gadolinium enhancement, *ECV* extracellular volume

**Table 4:** CMR findings systemic lupus erythematosus (SLE)

	Controls (*n* = 20)	Patients (*n* = 13)	*P*
LV-EF (%)	66 ± 4	63 ± 7	0.60
LV-EDV (ml)	109 ± 28	111 ± 36	1
LV-ESV (ml)	37 ± 13	43 ± 24	0.82
LV-SV	71 ± 16	67 ± 12	0.55
LV-EDD	45 ± 5	46 ± 4	1
LA (cm^2^)	20 ± 4	17 ± 3	0.02
IVS (mm)	10 ± 2	9 ± 1	0.35
PA (mm)	24 ± 4	22 ± 3	0.08
LV mass (g)	76 ± 18	85 ± 21	0.41
LGE per patient	-	3 (23 %)	
Epicardial	-	1 (8 %)	
Intramural	-	2 (15 %)	
Transmural	-	1 (8 %)	
Subendocardial	-	-	
% LV mass	-	6.2	
Native T1 (ms)	962 (947–987)	1002 (976–1015)	**0.03**
Post contrast T1 (ms)	526 (508–553)	507 (479–539)	0.16
ECV (%)	25 (24–27)	26 (25–29)	0.24
T2 (ms)	49 (48–51)	51 (49–53)	**0.02**

All values are n or mean ± SD or interquartile ranges

*CMR* cardiac magnetic resonance, *LV* left ventricular, *EF* ejection fraction, *EDV* end-diastolic volume, *ESV* end-systolic volume, *SV* stroke volume, *EDD* end-diastolic diameter, *LA* left atrium, *IVS* interventricular septum, *PA* pulmonary artery, *LGE* late gadolinium enhancement, *ECV* extracellular volume

### T1 and ECV results

**Fig. 1:**
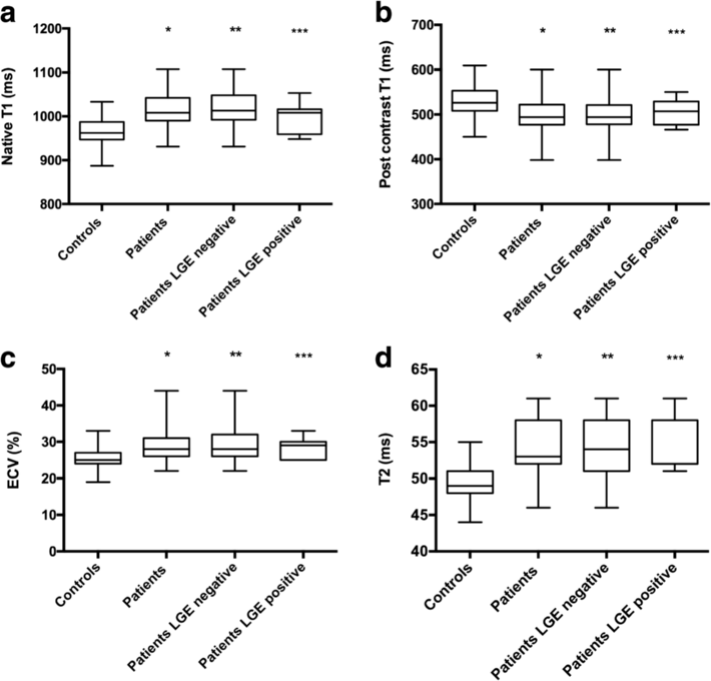
Box plots for median native T1 (**a**), post contrast T1 (**b**), extracellular volume fraction (ECV; **c**), and T2 mapping (**d**) in controls, all CTD patients, late gadolinium enhancement (LGE) negative CTD patients, and LGE positive CTD patients; the center line in each box represents the median, whereas the lower and upper limits of each box represent the 25th and 75th percentiles, respectively. CTD patients (all, LGE negative, LGE positive except for native T1 and post contrast T1, respectively) showed values which were significantly different to the values of the control group; */**/*** each *p* ≤ 0.05)

We found higher native T1 values in the CTD patient population: 1008 (990–1042) ms vs. 962 (947–987) ms in controls, *p* = 0.001; Table 2, Fig. 1a. Post contrast T1 values were decreased in comparison to controls: 494 (477–522) ms vs. 526 (508–553) ms, *p* = 0.008, Table 2, Fig. 1b. For T1-derived ECV measures, CTD patients demonstrated significantly higher values: 28 (26–31) % vs. 25 (24–27) % in the control group, *p* = 0.001, Table 2, Fig. 1c. LGE-positive CTD patients had no significant differences in their native, post T1 values, and ECV results as compared to LGE-negative CTD patients (*p* = 0.36, *p* = 0.63, *p* = 0.76, respectively).

**Fig. 2:**
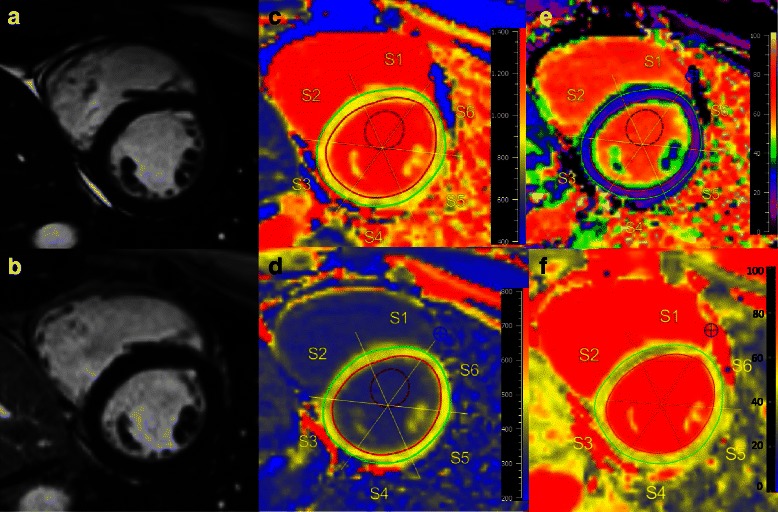
Patient with SLE. Cardiovascular magnetic resonance (CMR) of a 30-year old female presenting with no symptoms and normal ECG for her sixth cycle of cyclophosphamide. 3 months ago, the diagnosis SLE was settled. Cine images (**a**) revealed a preserved LV-EF (63 %), LGE images (**b**) demonstrated no enhancement. Native T1 map (**c**) showed an increased T1 with 999 ms (normal median range 962 (947–987) ms), decreased post-contrast T1 (**d**) with 489 ms (normal median range 526 (508–553) ms), and expanded ECV (**e**) of 27 % (normal median range 25 (24–27) %). T2 (**f**) was prolonged with 51 ms (normal median range 49 (48–51) ms)

Subgroup analysis of the SSc and SLE patients revealed higher T1 native and ECV values in SSc patients (1031 (1007–1075) ms, and 31 (28–34) %, respectively), compared to 1002 (976–1015) ms and 26 (25–29) % in SLE patients, both *p* < 0.01, also see Tables 3 and 4. Furthermore, post contrast T1 values were lower in SSc patients: 494 (474–525) vs. SLE patients: 507 (479–539). However, this difference was not statistically significant (*p* = 0.43). Compared to healthy controls, patients with SSc demonstrated: 1) significantly higher median native T1 values and ECV values (both *p* < 0.001), 2) significantly decreased post contrast values (*p* = 0.02). Patients with SLE showed increased median T1 native values in comparison to healthy controls (*p* = 0.03). However, although increased, ECV values did not differ significantly to controls (*p* = 0.24). Furthermore, SLE patients demonstrated lower post contrast values than controls without reaching significance (*p* = 0.16). Figure 2 displays a LGE-negative female SLE patient showing increased native T1, ECV and T2, and decreased post contrast T1 values.

In our patients with ECG abnormalities, native and post T1 values, and ECV were not significantly different to the values in patients with normal ECG (*p* = 0.73, *p* = 0.65, *p* = 0.93, respectively).

### T2 results

Median myocardial T2 values were significantly higher in patients with CTD than in controls: 53 (52–58) ms vs. 49 (48–51) ms, *p* < 0.001, Table 2, Fig. 1d. This difference remained significant independent of the patients’ LGE status; LGE-negative patients: 54 (51–58) ms, *p* ≤ 0.001, and LGE-positive patients: 52 (52–58) ms, *p* = 0.001.

In the subgroup analysis, SSc patients had higher T2 values than patients with SLE: 58 (54–59) ms vs. 51 (49–53) ms, *p* = 0.001. However, both (SSc and SLE) differed significantly to the control group, *p* < 0.001, *p* = 0.02, respectively, Tables 3 and 4.

**Fig. 3:**
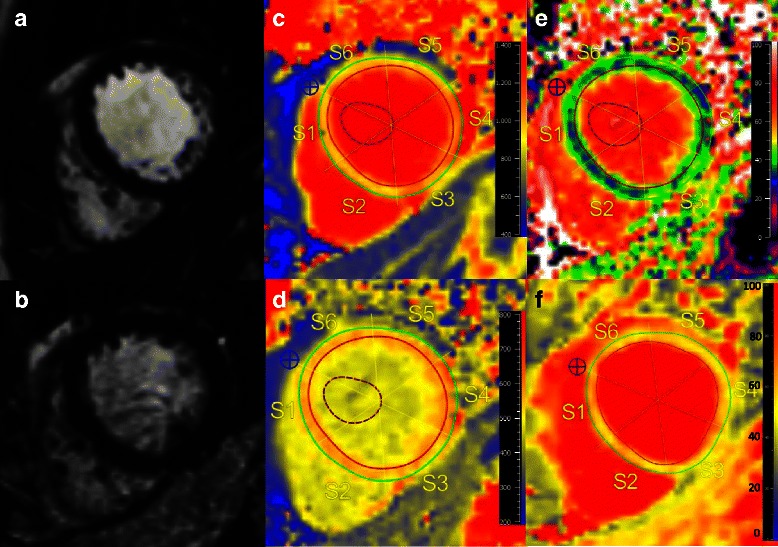
CMR of a 45-year old female with a history of SSc since 15 years. She was suffering from nonspecific dyspnea, and palpitations. ECG was unremarkable. Cine CMR (**a**) revealed a preserved LV-EF (61 %) with normal cardiac dimensions. LGE images (**b**) revealed no LGE. However, native T1 (**c**) with 1108 ms, ECV (**e**) with 40 %, and T2 (**f**) with 61 ms were severely increased, whereas post contrast T1 (540 ms) (**d**) was within normal range compared to our control group (median native T1 962 ms, ECV 25 %, post contrast T1 526 ms, T2 49 ms)

These findings are illustrated by Fig. 3, displaying a LGE negative SSc patient with severely increased values for native T1, ECV and T2, and decreased values for post contrast T1 in comparison to controls.

T2 values in patients with ECG abnormalities were not significantly different from T2 values in patients with normal ECG, *p* = 0.60.

### Values above the 95 % percentile of normal

**Fig. 4:**
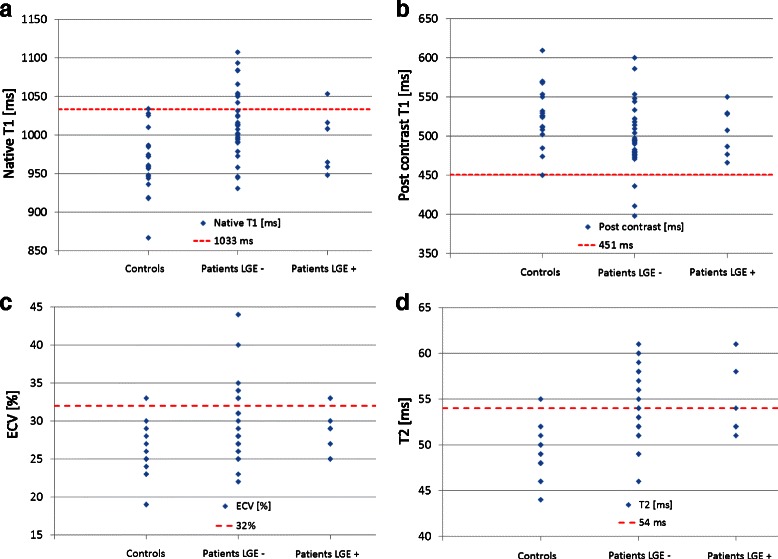
Values above the 95 % percentile of normal. Values for T1 (native (**a**), post contrast (**b**)), ECV (**c**) and T2 (**d**) in controls, LGE negative and LGE positive CTD patients indicating values beyond the 95th percentile (*dotted line*) of the control group, representing the threshold for definite abnormal values (1033 ms for native T1, 451 ms for post contrast T1, 32 for ECV, and 54 ms for T2). Interestingly, in LGE negative patients most frequent abnormal values were reported for native T1 (above 1033 ms), and for T2 (above 54 ms), suspicious of myocardial involvement. * = Some of the values might be similar, with dots overlapping one another

Defining the 95 % percentile of our control group as a threshold for definite abnormal values, we found values above 1033 ms for native T1, below 451 ms for post contrast T1, above 32 % for ECV, and above 54 ms for T2 to be abnormal, see Fig. 4.

25 % (*n* = 10) of the CTD patients demonstrated a native T1 value above the 95 % percentile of the matched control group: *n* = 9 patients were LGE-negative, *n* = 1 patient was LGE- positive, also see Fig. 4a. In *n* = 3 patients, post contrast values were below 455 ms, all of them were reported LGE-negative, see Fig. 4b. Measurement of ECV revealed that *n* = 8 patients had definite abnormal values (*n* = 7 LGE-negative, *n* = 1 LGE-positive), see Fig. 4c. Almost 40 % (15 out of 40 CTD patients) showed definite abnormal T2 values: *n* = 13 LGE-negative, *n* = 2 LGE-positive, see Fig. 4d.

**Fig. 5:**
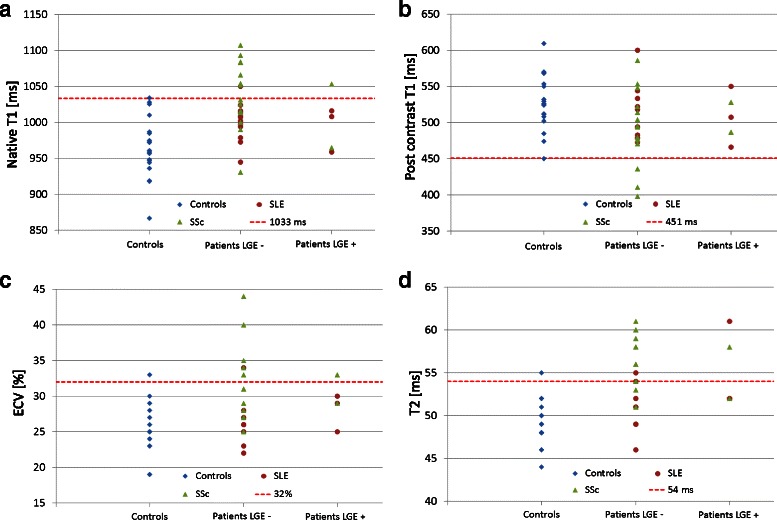
Subgroup analysis for patients with SSc and SLE. Values for native T1 (**a**), post contrast T1 (**b**), ECV (**c**) and T2 (**d**) in controls, LGE negative and LGE positive SSc/SLE patients, indicating values beyond the 95th percentile (*dotted line*) of the control group, representing the threshold for definite abnormal values (1033 ms for native T1, 451 ms for post contrast T1, 32 for ECV, and 54 ms for T2). * = Some of the values might be similar, with dots overlapping one another

Almost 50 % of SSc patients (8 out of 17 SSc) had an increased native T1 value above the 95 % percentile of controls, and only one of these was LGE-positive. Three patients (all LGE-negative) had post contrast T1 values lower, and six patients (5 LGE-negative, 1 LGE-positive) had ECV values higher than the 95 % percentile of controls. 65 % of the SSc patients demonstrated definitely abnormal T2 values: *n* = 10 were LGE-negative, *n* = 1 LGE-positive, also see Fig. 5.

In the SLE subgroup, values higher than the 95 % percentile of controls were found in *n* = 3 patients (23 %), with one patient showing both increased T2 and native T1 values beyond the 95 % percentile of normal, and the two other patients isolated increased ECV or T2 beyond the 95 % percentile of normal, respectively. Only one of these patients was reported LGE-positive, also see Fig. 5.

## Discussion

To the best of our knowledge, this is the first study evaluating cardiac involvement in patients with CTD and preserved left ventricular ejection fraction by a comprehensive CMR approach, including LGE CMR, as well as T1 and T2 mapping techniques. The findings are as follows: 1) Patients with CTD show increased native T1, ECV, T2 and decreased post contrast T1 values compared to controls. 2) Subgroup analysis of SSc and SLE patients revealed that native T1 and T2 values seem to be higher in patients with SSc compared to patients with SLE. However, both parameters can separate between SSc/SLE patients and controls. 3) Abnormal values beyond the 95 % percentile of healthy controls might help to detect myocardial involvement in patients with CTD even in the absence of LGE.

### Patient characteristics and general CMR results

Most patients were middle-aged and female, in line with previous reports [3]. The majority of patients was non- or oligosymptomatic, and had normal ECG, underlining that the diagnosis of cardiac involvement is a challenge in CTD, Table 1. The mean LV-EF in our cohort was preserved (62 %), cardiac dimensions did not differ from controls, Table 2. LGE was present in 18 % of the CTD patients, occurring in a non-ischemic pattern in accordance with other studies [2, 3, 20–22].

### T1 and ECV results

We found higher native T1 values and increased ECV in our CTD population in comparison to controls, Table 2, Fig. 1a + c. Furthermore, post contrast T1 values were decreased in comparison to controls, Table 2, and Fig. 1b. Since these differences are independent of the presence of LGE, they may allow early detection of subclinical myocardial alterations in patients with CTD, as reported in other inflammatory cardiomyopathies [23, 24].

In the SSc subgroup, differences for native T1 and ECV were even larger than in the overall CTD population, suggesting a high rate of diffuse myocardial involvement detected by T1 mapping, supporting data from Ntusi et al. [3], who found also elevated native T1 and ECV in SSc patients. At first sight, the mapping data in this study seem to conflict with the low prevalence of LGE (12 %). However, LGE has its strengths in detecting focal processes (e.g. infarcted myocardium vs. remote myocardium), whereas in diffuse processes this technique is of limited value. Conversely, mapping techniques, which provide absolute quantitative values, rather than just visual or semi-quantitative interpretation of the images, perform well in the assessment of diffuse myocardial processes [7]. Therefore, the T1 and ECV findings in this study might be the surrogate for the high rate of diffuse fibrosis (44–100 %) observed by endomyocardial biopsy or autopsy in SSc patients [25, 26], and might be a useful tool not only for detection of myocardial involvement, but also for evaluation of an adequate response to immunosuppressive agents during the clinical course of the disease.

In the SLE subgroup, we observed lower T1 and ECV differences to controls than in the SSc subgroup. Consequently, although showing increased ECV and decreased post contrast T1 values compared to controls, the difference was significant only for native T1 values, *p* = 0.03. This might have at least two reasons: 1) In contrast to SLE patients, autopsy studies from SSc patients revealed a high rate of diffuse fibrosis, which might be the surrogate for higher native T1 and ECV values in SSc patients [25, 26]. 2) Our finding that native T1 seems to separate best between SLE patients and healthy controls, is supported by a recent study [2], which identified native T1 a) as the best parameter to separate between SLE patients and controls, and b) as an independent predictor of the underlying SLE diagnosis. However, in the study by Puntmann [2] also post contrast T1 values and ECV differed significantly to the control group. They included 33 asymptomatic SLE patients, with an activity index (SLEDAI) of 0, and observed a high LGE prevalence of 61 % (*n* = 20), which is in contrast to our study (SLEDAI 16, prevalence of LGE 23 %). Another explanation for these differences might be the time duration from SLE diagnosis to CMR imaging: In the study from Puntmann et al., the average time from SLE diagnosis to imaging was 7.4 years whereas in our study almost 40 % had their CMR within the first year of SLE diagnosis. Therefore, it might be argued that the grade of diffuse fibrosis, as well as the presence of focal fibrosis detected by LGE, might increase in later stages of the disease. Since both studies found that native T1 is the most sensitive parameter to separate between SLE patients and controls, native T1 may play an important role in: a) initial diagnosis of myocardial involvement and b) the monitoring in SLE patients.

Our findings add knowledge to the potential role of T1 mapping in patients with different CTD, since this technique seems to provide more detailed tissue characterization than LGE alone. This might have clinical implications for the assessment of disease activity, and monitoring of the response to immunosuppressive medication in CTD patients. Moreover, since T1 and ECV values in patients with ECG abnormalities did not differ to the values of patients with normal ECG, the presence of ECG abnormalities alone may be of limited diagnostic value for detecting myocardial involvement in CTD patients.

### T2 results

In contrast to T1 mapping, myocardial T2 values correlate closely with free tissue water content [27, 28], predisposing them for the assessment of active myocardial inflammation in systemic disorders such as CTD. Newer T2 mapping sequences provide objective and robust data [8, 29], and will most likely replace previously described T2-weighted sequences [7].

As expected in systemic inflammatory disorders such as CTD, median myocardial T2 values were significantly higher than in controls, suggesting myocardial involvement due to systemic inflammation, Table 2, Fig. 1d. Of note, T2 performs even better than native T1 to separate controls from CTD patients (*p* < 0.001, *p* = 0.001, respectively). This difference remains significant by dividing the CTD population in a LGE-positive and a LGE-negative group, underlining the additional value of T2 mapping in comparison to the performance of LGE CMR alone.

For the SSc subgroup, we found only studies in the literature that used T2-weighted images for the assessment of inflammation instead of newer T2 mapping techniques [3, 30]. We filled this gap and found higher T2 values both than controls (*p* < 0.001), and patients with SLE (*p* = 0.001), suggesting a high grade of myocardial inflammation, possibly representing active disease, in SSc patients. The occurrence of both myocardial inflammation and diffuse fibrosis is a well-known finding in these patients [3]. Thus, a comprehensive CMR approach including LGE, T1 and T2 mapping seems a reasonable approach to evaluate both chronic and active stages of the disease in SSc patients.

Our data are also supported by a recent study [31], which reported elevated T2 values in SLE patients compared to controls. However, their T2 values were higher in SLE patients and controls as compared to the values in this study, which might have the following reasons: 1) Different patient populations: our patients were younger; 2) different grades of inflammation due to different immunosuppressive treatment regimen: 77 % of our patients were on steroids vs. only 17 % in the latter study. 3) Differences in the T2 mapping sequence and map analysis software. Therefore, as long as there are no consistent mapping sequences, each institution should create its individualized normal values [12]. Of note, T2 values of our control group were in line with the results of other groups [32].

Since increased T2 values are supposed to represent potentially reversible processes [31], T2 mapping might play an important role as a quantitative biomarker, which might serve as surrogate for response or failure of immunosuppressive agents.

As shown above for T1 values, T2 values in patients with normal vs. abnormal ECG did not differ significantly, underlining the need for further detailed tissue characterization for the detection of myocardial involvement in CTD.

### Values above the 95 % percentile of normal

Despite highly significant differences in T1 and T2 values between the CTD population and controls, there is still some overlap in values, hampering the diagnosis of myocardial involvement in the individual CTD patient, also see Fig. 1. Therefore, we used the 95 % percentile of our control group as a threshold for definite abnormal values in patients with CTD.

The majority of abnormal values were reported for T2 (*n* = 15), and native T1 (*n* = 10), suggesting to be the most promising parameters for potential detection of myocardial involvement. Of note, 87 % of these patients with elevated T2 values, and 90 % of the patients with elevated T1 native values, were LGE-negative, see Fig. 4.

In the SSc and SLE subgroups we found comparable results, with native T1 and T2 as most frequent parameters above the 95 % percentile of normal, and a high rate of LGE-negative patients, see Fig. 5. These findings underline the additional benefit of the newer mapping techniques compared to LGE imaging alone.

### Clinical implications

In this study, we could demonstrate that mapping sequences in addition to LGE-CMR might be useful for the detection of myocardial involvement in patients with CTD. Patients with CTD show higher T1, ECV, and T2 values compared to healthy controls. These findings are independent of the presence of LGE. Furthermore, subgroup analysis in SSc and SLE patients revealed that native T1 mapping and T2 mapping are the best parameters to separate between normal subjects and patients. This could be confirmed among patients with values higher than the 95 % percentile of controls, suggesting a combination of both fibrosis and inflammation in CTD patients.

Despite potential life-threatening complications by myocardial involvement of CTD, many patients will present with nonspecific symptoms, normal ECG, and preserved LV-EF. Thus, a comprehensive CMR approach may be of future clinical importance not only for detection of myocardial involvement but also for response to treatment. Nevertheless, larger randomized trials are warranted to investigate the diagnostic and prognostic value of abnormal mapping findings, before these sequences can be implemented in the clinical routine.

### Limitations

Several potential limitations need to be addressed. Due to the single center setting, potential center-specific bias cannot be excluded. However, since most mapping sequences are vendor and center specific, there is still a lack of established normal values and thresholds, so preferably centers should establish their own normal values and thresholds upon healthy controls, as suggested by current recommendations [12].

The overall CTD group, and in particular the SSc and SLE subgroups are small, but comparable in size to most of the studies in the current literature dealing with CTD. Furthermore, despite the relatively small numbers of patients, significant differences in the mapping parameters were measured compared to controls.

Measuring global myocardial T1 or T2 values in a single mid-ventricular slice might overlook focal processes. However, this approach is common practice [33, 34], less subjective and might be even better comparable to follow-up exams. Moreover, for comparing different CMR techniques (native T1, post contrast T1, ECV, T2), it is fundamental that measurements are made in matching locations.

Endomyocardial biopsy was not routinely performed. However, it is well known that EMB has several limitations, e.g. invasiveness, sampling error, lowering its diagnostic benefit. Furthermore, in oligosymptomatic patients with preserved LV-EF, this would be a rather unethical approach, and not in line with current guidelines [35].

Comparing mapping results to cardiac biomarkers would have been of interest, however this was not intention of our study, and should be investigated by further studies.

## Conclusions

We found increased values for native T1, ECV, T2, and decreased values for post contrast T1 in our CTD population with preserved LV-EF compared to controls, independent of the presence of LGE. Native T1, and T2 as the best discriminators to controls seem to have incremental value in the detection of myocardial involvement compared to LGE CMR alone, with the largest differences observed in patients with SSc. A potential benefit of the newer mapping techniques might be an early diagnosis of myocardial involvement in a still dynamic stage, yielding adequate treatment regimen, before irreversible scar (LGE) will manifest. Beside diagnosis, the new mapping techniques might be of value for monitoring of the disease.

However, further studies are mandatory, before the newer mapping sequences might be implemented in the daily clinical routine for decision-making in patients with CTD.

## Additional file

Additional file 1: Details of the used T1 mapping and T2 mapping sequences. (DOC 25 kb)

## Abbreviations

CMR: Cardiovascular magnetic resonance
CTD: Connective tissue disorders
CVD: Cardiovascular disease
ECG: Electrocardiogram
ECV: Extracellular volume
ICD: Internal cardioverter defibrillator
IQR: Interquartile range
IVS: Interventricular septum
LA: Left atrium
LGE: Late gadolinium enhancement
LV: Left ventricle
LV-EDV: Left ventricular end-diastolic volume
LV-EF: Left ventricular ejection fraction
LV-ESV: Left ventricular end-systolic volume
MOLLI: Modified look-locker inversion recovery sequence
SCD: Sudden cardiac death
SLE: Systemic lupus erythematosus
SSc: Systemic sclerosis
SSFP: Steady-state free-precession
