# Supplementary material for: Evaluation of myocardial involvement in patients with connective tissue disorders: a multi-parametric cardiovascular magnetic resonance study
Source: J Cardiovasc Magn Reson. 2016 Oct 13;18:67. doi: 10.1186/s12968-016-0288-4 (PMC5062828; doi:10.1186/s12968-016-0288-4)
Supplement: Additional file 1: — Details of the used T1 mapping and T2 mapping sequences. (DOC 25 kb) [file 12968_2016_288_MOESM1_ESM.doc]

**Appendix**

*CMR protocol*

**T1 Mapping**

T1 mapping was performed in short axis orientation using a modified Look-Locker inversion recovery (MOLLI) sequence before and 20 minutes after contrast media administration.

Three inversion episodes were employed, where in the first episode 3 images were acquired, followed by 3 recovery heartbeats, then 3 images in the second episode and another image in the 3^rd^ episode (3(3)3(0)1 MOLLI acquisition scheme), resulting in 7 images in total and a scan duration of 10 heartbeats.

Inversion times (TI) were 120, 200, 280 ms respectively in the initial images after inversion,

Typical imaging parameters were TE/TR 1.0/2.4 ms, acquisition time per heartbeat 168 ms, flip angle 35°, bandwidth 1371 Hz/pixel, matrix 192 × 136 pixels, measured in-plane spatial resolution 1.9 × 2.0 mm² and slice thickness 8 mm, PAT acceleration R=2.

**T2 Mapping**

T2 mapping was performed in short axis orientation before administration of contrast media using a T2-prepared single-shot bSSFP prototype sequence. T2-weighted images were obtained using ECG gating to diastole in a single breath-hold with 0, 25, and 55 ms T2 preparation times. Motion correction based on elastic registration was performed allowing generation of T2 pixel maps. After each single image acquisition, 4 heartbeats were used for signal recovery.

Typical imaging parameters were TE/TR 1.1/2.5 ms, acquisition time per heartbeat 150 ms, flip angle 70°, bandwidth 1445 Hz/pixel, matrix 192 × 116 pixels, measured in-plane spatial resolution 1.9 × 2.3 mm² and slice thickness 8 mm, PAT acceleration R=2.
